# Optimization of Selective Hydrolysis of Cruciferins for Production of Potent Mineral Chelating Peptides and Napins Purification to Valorize Total Rapeseed Meal Proteins

**DOI:** 10.3390/foods11172618

**Published:** 2022-08-29

**Authors:** Nastassia Kaugarenia, Sophie Beaubier, Erwann Durand, Arnaud Aymes, Pierre Villeneuve, François Lesage, Romain Kapel

**Affiliations:** 1 Laboratoire Réactions et Génie des Procédés, Unité Mixte de Recherche, Centre National de la Recherche Scientifique 7274, F-54500 Vandœuvre-lès-Nancy, France; 2 CIRAD, UMR QualiSud, F-34398 Montpellier, France; 3 Qualisud, Univ Montpellier, Avignon Université, CIRAD, Institut Agro, Université de la Réunion, F-34398 Montpellier, France

**Keywords:** rapeseed protein valorization, selective hydrolysis, modelling, economical optimization, metal-chelating peptides

## Abstract

Preventing oxidation and microbial spoilage are both major concerns in food industries. In this context, this study aimed to valorize the total rapeseed meal proteins with controlled enzymatic proteolysis to generate potent mineral-chelating peptides from cruciferins while keeping intact the antimicrobial napins. Implementation of proteolysis of total rapeseed protein isolate with the Prolyve^®^ enzyme highlighted an interesting selective hydrolysis of the cruciferins. Hence, the mechanism of this particular hydrolysis was investigated through a Design of Experiments method to obtain a model for the prediction of kinetics (cruciferin degradation and napin purity) according to the operating conditions applied. Then, multicriteria optimization was implemented to maximize the napin purity and yield while minimizing both enzymatic cost and reaction time. Antioxidant assays of the peptide fraction obtained under the optimal conditions proved the high metal-chelating activity preservation (EC_50_ = 247 ± 27 µg) for more than three times faster production. This fraction might counteract lipid oxidation or serve as preventing agents for micronutrient deficiencies, and the resulting purified napins may have applications in food safety against microbial contamination. These results can greatly help the development of rapeseed meal applications in food industries.

## 1. Introduction

Rapeseed is the second leading worldwide oilseed production seed with 73.1 million tons produced in 2018–2019, resulting in 19 million tons of meal, the solid residue remaining after the oil extraction process [1]. Rapeseed meal is commonly used as a feed supplement for livestock because of its high amount of proteins (from 30 to 50% on dry matter basis) [2]. Rapeseed proteins are distinguished in two major fractions: the 12S globulins, also called cruciferins, and the 2S albumins; also called napins [3]. Cruciferins and napins are storage proteins, synthesized during the seed’s embryonic development phase [4]. Napins represent from 13 to 46% of the total rapeseed protein content, and are low molecular weight proteins (12.7–21 kDa [5]) built from two disulfide-linked peptide chains with an isoelectric point around 11 [6,7,8,9]. Cruciferins constitute from 26 to 65% of the total protein content, and have high molecular weight (300–340 kDa [5]) with a hexamer structure combined by covalent and non-covalent bonds, with an isoelectric point of 7.2 [8,10].

Among the two main rapeseed protein fractions, napins are the most promising for food applications. Indeed, their interesting functional properties have been reported, such as foaming and emulsifying [11], as well as potential biological properties such as antifungal [12] and antimicrobial properties [13]. Napins have a well-balanced composition in amino acids, in accordance with the FAO/WHO/UNU recommendations of 2007 [14]. Hence, napins from rapeseed meal could be high added-value products. There are several possible ways to produce and valorize these proteins. The common one is a two-step process with total protein extraction followed by protein purification either by acidic precipitation or by ultrafiltration separation [15]. Nevertheless, these processes degrade the cruciferins, making them difficult to valorize in foods. Recently, it has been shown that napins could be selectively extracted upon acidic conditions [16,17]. This process also allows for the co-production of a high-quality solid residue rich in cruciferins and low in phytic acid, but only applicable for feed utilization.

Enzymatic hydrolysis can be an efficient way to increase the added-value of proteins with the release of bioactive peptides for example. Bioactive peptides are breakdown products of proteins obtained with proteases, which have specific biological function. Thus, biopeptides are defined as specific short protein fragments (2–20 amino acids) that have a positive impact on body function or condition and which may influence health [18]. Among them, antioxidative, ACE-inhibitory, anticancer, antimicrobial, or immunomodulating activities were reported [19,20,21]. Plant proteins constitute a great source of biopeptides and many have been produced from rapeseed proteins [22]. Attention has been given to the production of metal-chelating peptides either to prevent lipid oxidation in foods or to increase essential micronutrients absorption for nutritional and health purposes. A recent study highlighted a noteworthy metal-chelating activity of peptides (61.4 ± 5.90 µM of chelating iron (II)/mg peptide) from rapeseed meal proteins produced with the Prolyve^®^ enzyme [23]. This is all the more interesting since these peptides were produced mainly from the cruciferins resulting in intact napins fraction. Hence, this selective hydrolysis could lead to a double valorization of high added-value products for food safety applications with bioactive peptides from the cruciferins on the one hand and purified napins on the other. Nevertheless, this selective hydrolysis was implemented in a single enzymatic condition, without quantification of the napin purity and yield, nor optimization of the technical and/or economic criteria.

The aim of this study was to develop and implement an original methodology to model and optimize the selective hydrolysis of total rapeseed proteins with the Prolyve^®^ enzyme. To do so, three main steps were followed. The first one was the study of the selective hydrolysis mechanism as a function of sets of conditions that could most impact the enzymatic hydrolysis reaction (i.e., pH, temperature, and E/S ratio). The second step was the modelling of the kinetics reactions in the identified conditions range of the target mechanism, based on the recently published methodology of Beaubier et al. [24]. Kinetics of the cruciferins degradation and the napin purity were modeled. The last step was the optimization of the selective hydrolysis on technical and economic criteria, which are the reaction time and the enzymatic cost. Finally, the metal-chelating activity of the peptides obtained in the identified optimal conditions of selective hydrolysis were assessed to validate the preservation of this food application interest.

## 2. Materials and Methods

### 2.1. Materials and Chemicals

Rapeseed proteins isolate (RPI) was produced from a ground rapeseed meal provided by Olead (Pessac, France): the starting protein content in the meal was 34.4%, based on dry matter basis. Then, the extraction of total proteins was made from the meal with the same protocol described by Durand et al. [23]. The purity of the obtained extract (called RPI) was analyzed by Kjeldahl method with 6.25 nitrogen-to-protein conversion factor. It was measured at 92.8 ± 2.7% on dry matter basis, corresponding to an isolate grade. The initial napin and cruciferin purities into the RPI were determined by Size-Exclusion chromatography at 47.3% and 52.7%, respectively. This RPI was the substrate for all hydrolysis experiments.

The enzyme Prolyve^®^ (PAC 30 L), from *Aspergillus niger* with a specific activity of 585 UA/g, was purchased from Soufflet Biotechnologies (Nogent-sur-Seine, France). The optimum pH range was between 2.5 and 5.5. The optimum temperature ranged from 50 °C to 60 °C. The protease was food-grade and stored at 4 °C.

### 2.2. Enzymatic Proteolysis and Membrane Fractionation

#### 2.2.1. Batch Hydrolysis Experiments

Hydrolysis of RPI were carried out a in a 200 mL jacketed reactor. Temperature was water-bath controlled (Isotemp, Thermo Fisher Scientific, Waltham, MA, USA) and pH was maintained constant with a 902 Titrando system (Metrohm Ltd., Herisau, Switzerland) with 0.5 mol L^−1^ HCl. Initial RPI concentration was 1% (*w*/*v*, based on protein purity and dry matter basis). The suspension was driven to appropriate conditions (temperature and pH) prior to enzyme addition. 

The initial selective hydrolysis on RPI with the Prolyve^®^ enzyme was set at pH 3.0, 50 °C, and E/S ratio of 1/500 (g enzyme/g substrate). The final volume was 50 mL. The reaction was stopped after 7 h of hydrolysis by a pH-shift from 3 to 9. 

For the mechanistic study, a suspension of RPI was implemented in a final volume of 60 mL. Temperature and pH range were according to supplier’s data: 40–50–60 °C and pH 3.0–4.0–5.0. The applied E/S ratio range was 1/100–1/500–1/1000 (g enzyme/g substrate). Samples (1 mL) were collected at 0–0.25–0.5–1–3–5 h. 

#### 2.2.2. Ultrafiltration of Prolyve^®^ Hydrolysates

Peptides and unhydrolyzed proteins constituting the RPI hydrolysate obtained with Prolyve^®^ were separated by ultrafiltration process by a volumetric concentration factor of 2, followed by a diafiltration with 5 diavolumes of ultrapure water. A regenerated cellulose 3 kDa membrane was used (88 cm², Millipore, Burligton, MA, USA), on a Cogent^®^ µScale TFF system (Millipore, Burlington, MA, USA) at room temperature and 2.5 bar of transmembrane pressure. Permeate was collected, concentrated about 5 times with Rotavapor Büchi (Marshall Scientific, Hampton, NH, USA) at 45 °C for 5 h, and freeze-dried.

### 2.3. Modelling and Optimizing Methodology

#### 2.3.1. Simulation Methodology

Beaubier et al. [24] made assumptions that the protein conversion rate (*Xp*) followed second order kinetic reaction, based on the work of Deng et al. [25]. It was assumed that the same strategy could be assigned to modelling the protein concentration kinetic. Indeed, the *Xp* is the relation of remaining protein concentration $\left(Cm_{t}\right)$ to the initial protein concentration ($Cm_{0})$, as follows (Equation (1)):

(1) $$Xp_{t}=\left(1-\frac{Cm_{t}}{Cm_{0}}\right)$$

Then, the parameter $Xp_{max}$ can be defined as the maximum amount of initially inserted proteins that can be hydrolyzed in the conditions applied. Hence, a minimum protein concentration ($Cm_{min}$), which would not be hydrolyzed, can be determined, and $Xp_{max}$ can be calculated as (Equation (2)):

(2) $$Xp_{tmax}=\left(1-\frac{Cm_{min}}{Cm_{0}}\right)$$

The kinetic of protein concentration during hydrolysis can therefore be modelled according to the following equation (Equation (3)):

(3) $$Cm\left(t\right)=\left(\frac{Cm_{min}}{Cm_{0}}+\frac{1-\frac{Cm_{min}}{Cm_{0}}}{1+\left(1-\frac{Cm_{min}}{Cm_{0}}\right)\times k_{C}\times t}\right)\times Cm_{0}$$

with $k_{C}$, the kinetic parameter for protein concentration variation.

#### 2.3.2. Kinetics Modelling

Prolyve^®^ proteolysis kinetics were therefore modelled according to Beaubier et al. [24]. A central composite face-centered design of experiment (DoE) was performed to build the kinetic model of the constant $k_{C}$ as a function of operating conditions T and E/S. These two factors have 3 coded levels (Table 1). The matrix consisted of 12 experiments whose 3 were the replicate of the center point. The response $k_{C}$ (*Y*) was obtained by regression of the modified second order kinetic (Equation (3)). Correlation models were polynomial equations (Equation (4)) with two operating conditions as variables (T and E/S) and intercept ($b_{0}$), linear ($b_{i}$), interaction ($b_{ij}$), and quadratic coefficient ($b_{ii}$):

(4) $$Y=b_{0}+\overset{3}{\underset{i=1}{\sum }}b_{i\cdot }X_{i}+\overset{3}{\underset{i=1}{\sum }}b_{ii\cdot }X_{i}^{2}+\overset{3}{\underset{i<j=1}{\sum }}b_{ij\cdot }X_{i}\cdot X_{j}$$

Extra kinetic prediction experiments were realized to confirm the kinetic modelling following the indications in Table 2.

MATLAB^®^ (R2020a, MathWorks, Natick, MA, USA) was used to fit the experimental data to the polynomial equation. An ANOVA was applied to evaluate the statistical significance of these model coefficients. Final correlation models were obtained by suppressing the non-significant terms (*p*-value > 0.05). The coefficients R^2^ and Q^2^, the relative standard deviation (RSD), the reproducibility and the lack-of-fit were analyzed to characterize the model goodness-of-fit. 

#### 2.3.3. Multicriteria Optimization

The multicriteria optimization of the enzymatic proteolysis process was implemented on technical and economic criteria. The optimum operating conditions for the production of target hydrolysate were thus sought in order to minimize two performance criteria: the reaction time and the enzymatic cost. A MATLAB^®^ program employing a genetic-evolutionary algorithm developed in the laboratory [26] was used to identify the front and Pareto domain. A population of 2000 individuals was randomly generated by the program.

Two main objective functions were set to minimize the enzymatic cost (in € per kg of proteins) and the time (h) of hydrolysis based on napin purity. To do so, cruciferin concentration was implemented, determined from the kinetic model obtained as a function of hydrolysis operating conditions (Equation (4)). Napin yield was set at 94% and purity was fixed at 84% (maximum experimentally reached). Among all possible solutions, the single best trade-off was chosen by the “min-max” method [27].

### 2.4. Analytical Methods

#### 2.4.1. Protein Analysis and Quantification

The obtained hydrolysates were analyzed according to the methodology described by Defaix et al. [28]: 5 µL of sample were injected onto a Biosep-SEC-s2000 300 × 7.8 mm column, 5 µm (Phenomenex, Torrance, CA, USA) kept at 35 °C, connected to a Shimadzu model LC20 system (Shimadzu Corporation, Kyoto, Japan). An isocratic elution was used to separate the samples at 0.6 mL min^−1^ with a water/acetonitrile/trifluoroacetic acid (TFA): 54.9/45/0.1 (*v*/*v*/*v*) solvent. UV signal was recorded at 214 nm using a cell with an optical path of 1 cm.

The hydrolysate exploitation was based on SE-HPLC quantification [28]. Briefly, protein peak integration was converted to protein mass concentration through the Beer–Lamber law by approximating the unknown mass extinction coefficient ($\varepsilon_{Prot}$) as previously described [29]. Protein mass concentration was determined as following (Equation (5)):

(5) $$Cm_{Prot}=\frac{Q}{V_{inj}\times l}\times \overset{RT_{fin}}{\underset{RT_{ini}}{\int }}\frac{A_{Prot}}{\varepsilon_{Prot}}\cdot dRT$$

where *Q* is the flow rate, $V_{inj}$ the injected volume, *l* the length path, $A_{Prot}$ the intensity signal at 214 nm of protein, $\varepsilon_{Prot}$ the mass extinction coefficient of protein and *RT* the retention time. 

The mass extinction coefficient of protein was given by (Equation (6)):

(6) $$\varepsilon_{Prot}=\frac{\varepsilon_{liaison}\times n_{peptite\;bond}+\sum_{i=1}^{20}\varepsilon_{amino\;acid}\times n_{amino\;acid}}{MM_{Prot}}$$

The molar mass of proteins $MM_{Prot}$ were taken from available UniProt databases: BnC1 from *Brassica napus* [UniProt-P33523 (CRU1_BRANA)] for cruciferins and Napin-3 from *Brassica napus* [UniProt-P80208 (2SS3_BRANA)] for napins.

Yield and purity of napins were calculated from following equations (Equations (7) and (8)):

(7) $$Yield_{Nap}=\frac{Cm_{Nap}\left(t\right)}{Cm_{Nap}\left(t=0\right)}$$

(8) $$Purity_{Nap}=\frac{m_{Nap}\left(t\right)}{m_{Nap}\left(t\right)+m_{Nap}\left(t\right)}$$

where $m_{Nap}$ is the mass (g) of napin.

#### 2.4.2. Peptide Analysis and Quantification

Hydrolysate quantification was monitored by SE-HPLC analyzes according to Beaubier et al. [30]. A Superdex peptide 10/300 GL column (10 × 300 mm, GE Healthcare, USA) was used, kept at 35 °C, and connected to a Shimadzu model LC20 system (Shimadzu Corporation, Kyoto, Japan). A 10 µL sample was injected and eluted with an isocratic elution at 0.5 mL min^−1^ with a water/acetonitrile/trifluoroacetic acid (TFA): 69.9/30/0.1 (*v*/*v*/*v*) solvent. An optical path of 0.5 cm was used to record UV signal at 214 nm. Eleven synthetic peptide standards with MW ranged from 220 to 1890 g/mol were used to do the column calibration.

Degree of hydrolysis (*DH*) and protein conversion rate (*Xp*) were calculated according to the previously developed methodology [30]. Briefly, the amount of hydrolyzed proteins was evaluated by comparing the protein signal at time *t* to the initial protein signal, as follows (Equation (9)):

(9) $$Xp_{t}\left(\%\right)=\left(1-\frac{A_{t}}{A_{0}}\right)\times 100$$

where $Xp_{t}$ is the protein conversion rate at a given time *t* of the reaction, $A_{t}$ is the protein absorbance signal at time *t*, and $A_{0}$ is the initial protein absorbance without enzyme. 

According to Adler-Nissen [31], the degree of hydrolysis definition is the percentage of the total number of peptide bonds in a protein which have been cleaved during hydrolysis. It can thus be calculated from the parameters $Xp_{t}$ and the mean number of amino acids by peptide $\bar{Naa}$, according to Equation (10) and Beaubier et al. [30]:

(10) $$DH_{t}\left(\%\right)=\frac{1}{\bar{Naa}}\times Xp_{t}\left(\%\right)$$

### 2.5. Iron (II) Chelating Activity

The method used was the same as described by Durand et al. [23]. Chelating capacity was measured according to the following Equation (11):

(11) $$Chelating\;rate\;\left(\%\right)=\frac{A_{0}-\left(A_{1}-A\right)}{A_{0}}\times 100$$

where $A_{0}$ was the absorbance of the control (blank FeCl_2_ and Ferrozine without sample), $A_{1}$ the absorbance of sample without reactant, and A the sample absorbance with FerroZine™ reactant. 

Results expressed as µM iron (II) chelating/mg of sample, were exploited by the linear relationship equation of the total chelated iron (II) at different sample concentrations. Results as EC_50_ were expressed as the quantity (in µg) of sample that is required to chelate 50% (~7.9 µg) of iron (II) from FeCl_2_.

### 2.6. Statistical Analysis

Chelating assays were made in triplicate. Variance analysis and multiple comparison test were made with MATLAB© (R2020a, MathWorks Inc., Natick, MA, USA). Statistical analysis for two-samples with *t*-test was performed using Microsoft Excel 2016 (Microsoft Corporation, Redmond, WA, USA). Significative difference was considered at *p*-value < 0.05.

## 3. Results

### 3.1. Study of the Selective Hydrolysis Mechanism of Rapeseed Proteins

#### 3.1.1. Highlighting of Selective Hydrolysis of Cruciferins

**Figure 1:**
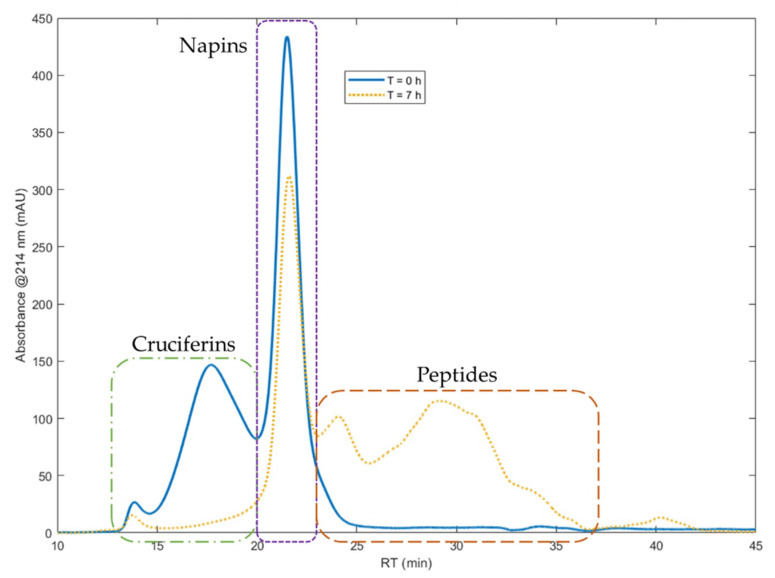
Size exclusion chromatograms of total rapeseed protein isolate at time *t* = 0 (blue curve) and at time *t* = 7 h of hydrolysis reaction with the protease Prolyve^®^ (yellow curve). Chromatographic system: Superdex Peptide 10/300 GL column, detection at 214 nm, solvent: water/acetonitrile/TFA (69.9/30/0.1), flow rate of 0.5 mL min^−1^.

Isolate of total rapeseed meal proteins (RPI) was hydrolyzed with the industrial acid protease Prolyve^®^, in the recently reported reaction conditions [23], i.e., at pH 3, 50 °C and E/S of 1/500, during 7 h. Figure 1 shows SEC chromatograms obtained for RPI before hydrolysis and after 7 h hydrolysis.

The first peaks of the RPI SEC chromatogram correspond to cruciferins, followed by the peaks of napins [28]. Cruciferins signal extends from 12.4 to 19.7 min and napins signal from 19.7 to 22.5 min of retention time. Considering the column calibration, peptides begin at 22.5 min and end at 39.2 min, beyond which there is free amino acids signal. The SEC results clearly showed that the cruciferins were almost totally depleted with Prolyve^®^ enzyme, unlike the napins which were hardly hydrolyzed. In these hydrolysis conditions, a *DH* value of 7 ± 0.3% was determined for the hydrolysate obtained after 7 h. The napin purity (representing the napin concentration on total protein concentration) and the napin yield (corresponding to the napin concentration at time “*t*” on initial concentration) were quantified as 86.6 ± 1.7% (compared to 47.3% in the initial protein isolate) and 84 ± 0.2%, respectively. Hence, more than 80% of napins were left intact and the hydrolysate mixture was enriched in napins by a factor of 1.5. 

This interesting selective hydrolysis of one protein in a complex mixture under acidic conditions was also reported with whey proteins. Indeed, at pH from 1.5 to 3.0, acidic enzymes (pepsin, protease A (*Aspergillus niger*) and protease M (*Aspergillus* sp.)) could selectively hydrolyze α-lactalbumin (α-La) whilst leaving β-lactoglobulin (β-Lg) intact. β-Lg is a well-known gastric digestion resistant protein, because of its four disulfide bridges, its hydrophobicity and its stability, higher at acid pH [32,33]. It was explained by the enhancement of ionization with decreasing pH and so the internal hydrogen bonds which are increased between amino-acids side chains [33,34]. Napins showed similar resistant properties toward pepsin and even trypsin hydrolysis [35,36]. They also have four disulfide bridges and showed minimal secondary structure changes and thermostability at pH 3.0 [37,38]. Moreover, napins have been shown to be partially unfolded at pH 3.0, exposing a surface 12 times more hydrophobic than at pH 7.0 [37,39]. Hydrophobic residues at the protein surface may contribute to an enthalpy-driven stabilization [40].

This selective proteolysis of RPI highlighted in this study has a potential for the coproduction of bioactive peptides from cruciferins (high metal-chelating activity reported [23]) and intact napins, proteins with important technical, functional, and antimicrobial properties [12,41,42]. For further investigation, membrane fractionation (ultrafiltration process with 3 kDa membrane) was implemented to remove cruciferin peptides in permeate while purifying napins in retentate. The membrane selectivity was good for peptides less than 3 kDa (data not shown) and the metal-chelating activity of permeate was tested. The activity of this purified fraction of cruciferin peptides was four times more effective (EC_50_ = 276.5 ± 6.2 µg) than that of the whole hydrolysate. Nevertheless, the impact of hydrolysis reaction conditions on hydrolysis of RPI under acid pH was neither analyzed nor optimized.

#### 3.1.2. Impact of Reaction Conditions on the Selective Hydrolysis Mechanism 

**Figure 2:**
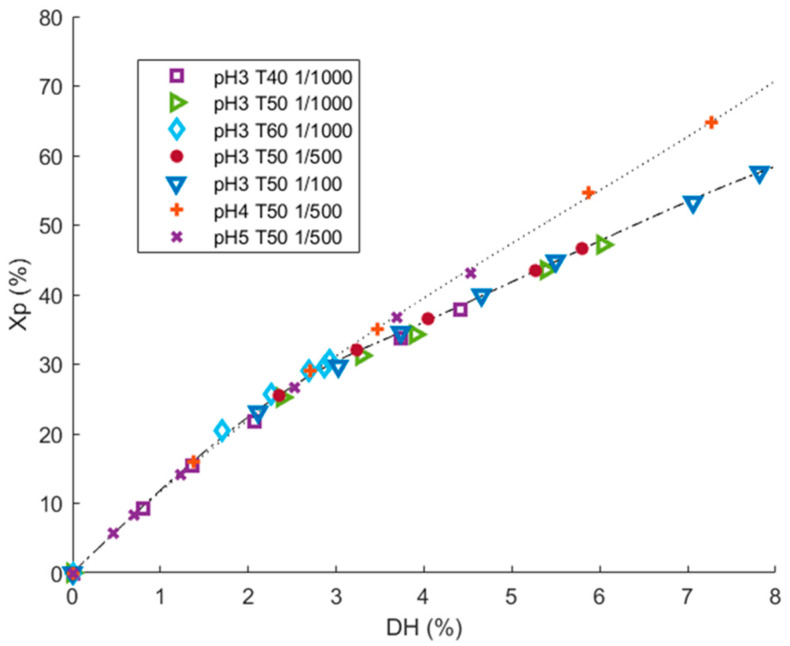
Identification of enzymatic mechanism for the hydrolysis of 1% (*w*/*v*) rapeseed proteins with Prolyv^®^ in the operating conditions area applied, during 5 h, by plotting the protein conversion rate (*Xp*) values versus degree of hydrolysis (*DH*) values.

There are two different kinds of proteolysis mechanisms: one-by-one and zipper [43]. Under the one-by-one mechanism, proteins will be progressively hydrolyzed quickly into a set of peptides of constant composition. In the zipper mechanism, proteins are quickly hydrolyzed into a fraction of large peptides that will be hydrolyzed themselves into a fraction of final peptides [43]. Proteolysis operating condition like pH, T, or E/S ratio are known to both impact proteolysis kinetics (*DH* = f(*t*)) and hydrolysis mechanism. Recently, Beaubier et al. showed that the impact of operating conditions on proteolysis mechanism can be elucidated by the trend of protein conversion rate (*Xp*) as a function of *DH* plots [24,30]. Figure 2. shows *Xp* = f(*DH*) plots at pH ranging from 3.0 to 5.0, temperature ranging from 40 to 60 °C, and E/S ranging from 1/100 to 1/1000 (g enzyme/g substrate). These conditions were chosen according to supplier recommendations and correspond to a zone of significant proteolysis activity.

The same hydrolysis mechanism was observed from *DH* 0 to 3%, corresponding to a protein conversion rate of 30%. Beyond *DH* 3%, two mechanisms were clearly observed in the applied condition domain. The two mechanisms seemed influenced by the pH value (pH 3.0 vs. 4.0 and 5.0). This was suggested by the fact that at a given *DH* value, two different *Xp* values can be achieved according to the pH applied (as an example, at *DH* 6%, *Xp* was around 44% at pH 3.0 and around 52% at pH 4.0). The depletion of the proteins was less linear at pH 3.0 than pH 4.0 and 5.0. Hence, two distinct enzymatic mechanisms can be observed in the applied conditions range, controlled by the pH value. Moreover, for set pH value (3.0) and temperature (50 °C) at same hydrolysis duration (5 h), hydrolysis with higher E/S (1/100) reached higher *DH* (*DH* 8% vs. 6%). For set pH value (3.0) and E/S ratio (1/1000) at same hydrolysis duration (5 h), *DH* values were higher at 50 °C (6%) than at 40 °C (4.5%) and at 60 °C (3%). Hence, the applied temperature and E/S ratio influenced the hydrolysis kinetics, but not the hydrolysis mechanism. These two conditions are evident and well-known kinetic parameters.

**Figure 3:**
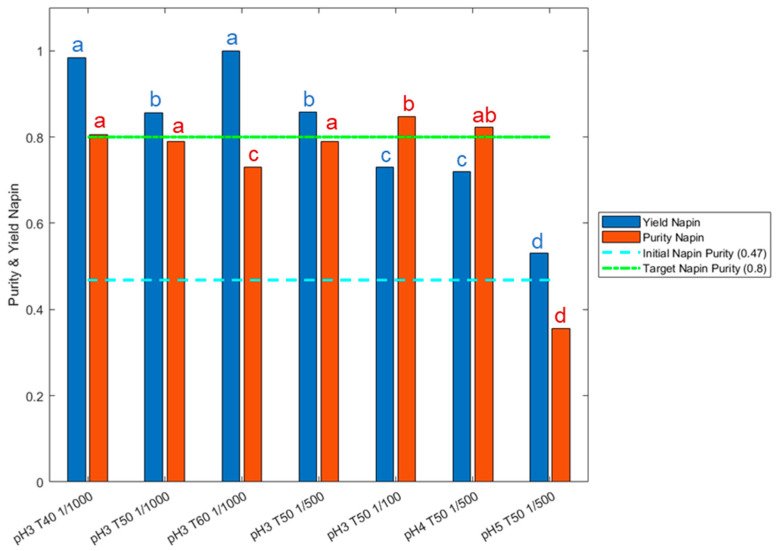
Napin purity (red bar and letters) and napin yield (blue bar and letters) quantification for hydrolysates obtained with Prolyve^®^ at 1% total rapeseed proteins (*w*/*v*) at pH range from 3.0 to 5.0, temperature from 40 to 60 °C and E/S ratio from 1/100 to 1/1000, after 5 h of hydrolysis. Statistically equivalent levels are under same letter.

These previous results reflected impact of operating conditions on total RPI hydrolysis kinetics, but not on napin purity and yield. Figure 3 thus exposes these criteria quantified by SEC for the hydrolysate obtained after 5 h in the previous applied conditions. Initial napin purity in the RPI was 47.3% and a napin yield of 80% (i.e., 20% maximum of napin depletion) was targeted. The results clearly demonstrated napin depletion at pH 4.0 and above, with napin yield below 80%. This is in accordance with the mechanism trend observed in Figure 2 at these pH values, with higher *Xp* values. Napin purity was very low at pH 5.0 (35.6%), but high at pH 4.0 (82.2%), and significantly equivalent at pH 3.0. This is explained by a simultaneous hydrolysis of both RPI fractions at pH 4.0 but a preferential hydrolysis of the napins at pH 5.0. The same observation was made with whey proteins, where a pH value above 4.0 was required to deplete β-Lg [44]. The results also showed an influence of the temperature and E/S ratio on the napin depletion. At pH 3.0, 50 °C seemed to be the optimal temperature for napin hydrolysis with Prolyve^®^ in the applied conditions. Furthermore, a low E/S ratio (>1/100; g enzyme/g substrate) was required to maintain high napin yield (>80%). 

### 3.2. Modelling of the Kinetic Reactions of Selective Hydrolysis

The second step to optimize the selective RPI proteolysis was the modelling of cruciferin degradation kinetics and the napin purity and yield. This modelling part was carried out with the implementation of a Design of Experiments (DoE) in the previous identified domain of operating conditions to preserve the selective hydrolysis of cruciferins. The experimental design was thus defined at pH 3.0 (constant value) with a temperature range chosen between 35 and 55 °C (to stay under the thermal destabilization of Prolyve^®^) and E/S ratio varying from 1/154 to 1/2000 (g enzyme/g substrate), in order to have significant enzyme concentration without a harmful excess of enzyme toward napins.

DoE was executed to establish correlations between the kinetic constants $k_{C}$ of both cruciferin concentration and napin concentration, with two operating conditions (T and E/S) at constant pH of 3.0, as described in a recently published methodology [24]. The application of this methodology would thus allow modelling the concentrations of both cruciferins and napins at any time of the hydrolysis reaction, as well as calculation of the corresponding napin purity and yield.

For the kinetic constant $k_{C}$ of the napin concentration kinetics, a non-significant model was highlighted with ANOVA analysis (*p*-value > 0.05). The operating conditions had no impact on the napin hydrolysis kinetics, which is explained by the fact that they were not hydrolyzed in the applied domain (pH 3.0). A significant model was achieved for the prediction of the kinetic constant of the cruciferin concentration ($k_{C}$). Table 3 shows regression coefficients of predicted quadratic polynomial models for $k_{C}$ of cruciferins concentration kinetics yielded by DoE. The intercept ($b_{0}$), linear ($b_{1}$ and $b_{2}$), quadratic ($b_{4}$ and $b_{5}$), and interaction ($b_{3}$) coefficients are presented. 

A robust linear fit regression model ‘fitlm’ from MATLAB^®^ was used to analyze the model correlation between the cruciferin kinetic constant $k_{C}$ and the operating conditions. Linear terms ($b_{1}$, $b_{2}$) and interaction ($b_{3}$) have been found to have a high influence on the response. The quadratic term ($b_{4}$) of the E/S was not significant. The correlations between the experimental data and the model had high coefficients of determination: R² = 0.99 and Q² = 0.96, meaning that the model for $k_{C}$ prediction fitted the experimental data well and was robust to predict new data.

**Figure 4:**
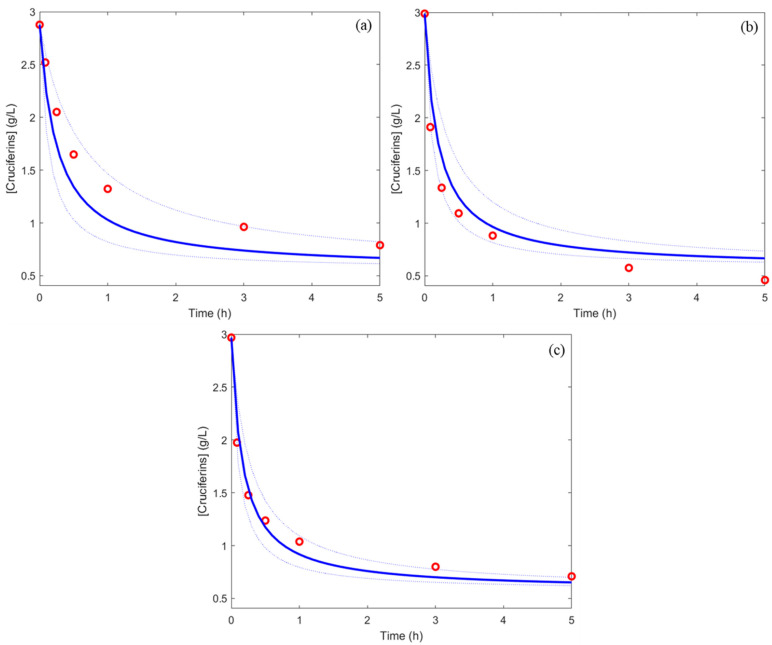
Experimental concentration (red points) and predicted concentration (blue line) with 95% predicted intervals (blue pointed line) for three different validation experiments (**a**–**c**).

Three new sets of conditions (a, b, and c) in the DoE matrix (Table 2) were implemented to validate the simulation of cruciferin concentration kinetics. Figure 4 shows the predicted kinetics from the obtained model and experimental kinetics for these conditions. All the experimental kinetics (a, b, and c) fitted well to the kinetics predicted by the model. This meant that the obtained model was significant, and the prediction accuracy was acceptable. Finally, this model can be used to predict hydrolysis kinetics reliably whatever the operating conditions.

**Figure 5:**
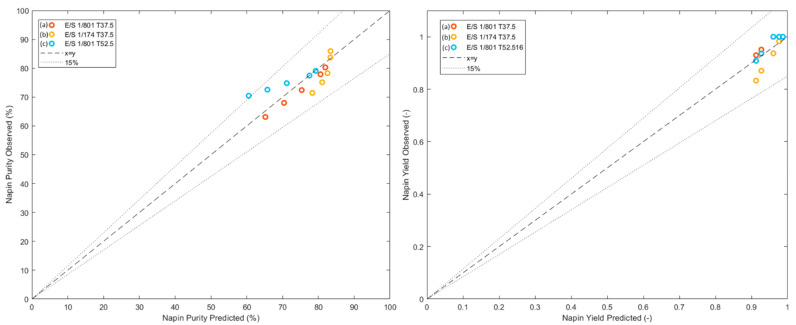
Correlations between experimental and predicted values of napin purity (**left**) and yield (**right**) for predicted kinetics (a, b, and c), and 15% error interval.

From the obtained model, Equations (10) and (11), the napin purity and yield were predicted for the three sets of conditions and compared to experimental values. Figure 5 shows the experimental versus predicted values of these two criteria. The results exposed good prediction of these criteria with R² between 0.85 and 0.99 for all validations points, which validated the applicability of the methodology and the model thus obtained in the case of selective hydrolysis of RPI with Prolyve^®^ enzyme.

### 3.3. Multicriteria Optimization of the Selective Hydrolysis

#### 3.3.1. Search for the Best Duration/Enzymatic Cost Trade-Offs

The last step to optimize the selective hydrolysis of RPI is to implement the validated kinetic model in a multicriteria optimization tool. Indeed, the main industrial criteria to be optimized for enzymatic hydrolysis are the enzymatic cost and the reaction duration, which are antagonist. Hence, the best reaction duration/enzymatic cost trade-offs have to be identified in this case of selective hydrolysis. To do this, a program developed on MATLAB^®^, which uses a diploid genetic-evolutionary algorithm exploiting the Pareto’s domination concept, was applied [26]. This tool crosses the domain of operating conditions and identifies the Pareto’s front and domain. The Pareto’s front represents the set of all non-dominant solutions, i.e., all acceptable duration/enzymatic cost trade-offs, and the corresponding operating conditions are represented in the Pareto’s domain. The reaction duration (*t*) was isolated from the model equation of cruciferin concentration determination (Equation (5)), which was placed in the napin purity equation (Equation (11)). The enzymatic cost was calculated knowing the E/S ratio required and the enzyme price per kg of substrate (fixed value of 35 € kg^−1^ of enzyme from the supplier). The objective functions were therefore programmed to be minimized (hydrolysis time and enzyme cost) with targeted napin purity at 84% and yield fixed at 94%.

**Figure 6:**
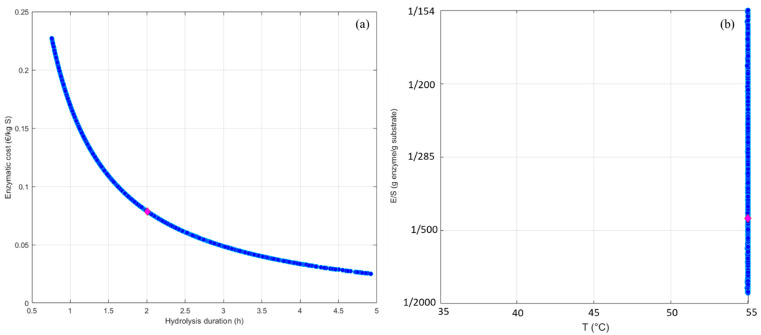
Identification of the Pareto’s front (**a**) and the Pareto’s domain (**b**), (blue points) and selection of the best duration/enzymatic cost trade-off (pink diamond) in the case of the applied study. Enzymatic cost is expressed in € per amount of protein (kg substrate).

Figure 6 shows the Pareto’s front obtained and the corresponding Pareto’s domain in the case of RPI hydrolysis with Prolyve^®^. A unique trend was highlighted where a decrease in the enzymatic cost implied an increase in the hydrolysis duration. The Pareto’s front exposed the optimal combinations of T and E/S (set pH value) for each acceptable trade-off, in a two-dimensional space. All acceptable trade-offs covered the E/S range of the applied domain, which was explained by the fact that this parameter is involved in the calculation of the enzymatic cost performance criterion. Interestingly, all solutions were found at the same temperature (55 °C), which was the maximal temperature applied in the optimization. This meant that the temperature was not a discriminant operating condition. This was in accordance with previous work [24], which also highlighted the fact that the best reaction duration/enzymatic cost trade-off should be searched by tuning the E/S ratio in the set pH value and at optimal temperature (55 °C in this work).

The most appropriate reaction duration/enzymatic cost trade-off can be then chosen among all solutions. To do this, many methods can be used to help with the decision. One of the simplest is the “min-max” solution. The purpose is to find the location of the solutions from functions such that its maximum deviation is minimized, i.e., that gives a solution which represents of the “center” of the Pareto front [27]. In this present case, the best solution was thus found for a hydrolysis time of 2 h, which is compatible with industrial implementations. This trade-off can be achieved at 55 °C with an E/S ratio of 1/448 (g enzyme/g substrate) and implied an enzymatic cost of 0.078 €/kg of substrate. These identified conditions allowed an optimization of the selective RPI proteolysis with Prolyve^®^. Indeed, the initial conditions implemented randomly were an E/S ratio of 1/500 (g enzyme/g substrate), a temperature of 50 °C and a reaction duration of 7 h. Hence, the enzymatic cost was almost equivalent (about 10% difference) but the reaction duration was reduced by a factor of 3.5, making it possible to reach the target hydrolysate very quickly.

These optimal conditions were applied experimentally (n = 4) and resulted in a napin purity of 80.1 ± 2.1% and a yield of 95 ± 2.5%. Therefore, this represents 4.6% and 1.1% of difference, respectively, compared to the targeted values (84% napin purity and 94% napin yield), validating the optimization carried out.

#### 3.3.2. Validation of the Bioactivity Preservation in Optimized Hydrolysis Conditions

The last step was the validation of the preservation of the important metal-chelating activity of the peptides produced by selective hydrolysis of cruciferins under the identified optimized conditions. Ultrafiltration of the optimized hydrolysate was performed and the metal-chelating activity of the resulting peptide fraction was determined with the Ferrozine assay as described in the material and methods section. Sodium citrate and EDTA salt, which are two of the most well-known metal chelators used in the food industry to avoid lipid oxidation, were also analyzed for comparison. The peptide fractions obtained under initial and optimized conditions have the same chelating efficiency with EC_50_ around 250 µg of sample (expressed as the quantity required to chelate 50% (~7.9 µg) of iron (II) from FeCl_2_), and were not statistically different (at *p* < 0.05). They were between the two commercial references, with EDTA demonstrating higher chelating efficiency (EC_50_ around 20 µg), and sodium citrate exhibiting lower efficiency (EC_50_ around 390 µg).

Results proved that the interesting metal-chelating activity was preserved with the peptides obtained under the optimized conditions. This was all the more interesting since the hydrolysis was 3.5 times faster and implemented with an enzymatic cost reduced by 1.5 times, without making any change in the chelating efficiency of the peptides thus obtained and maintaining a high napin purity.

## 4. Conclusions

Metal-chelating activity of biopeptides, particularly from biomass products, have great importance in the replacement of controversial industrial food preservatives. The present study exploited and investigated the original particularity of the selective hydrolysis of cruciferins from total rapeseed proteins with the Prolyve^®^ enzyme, leading to potent iron-chelating peptides. A complete approach was employed to optimize the production towards technical and economic criteria. This included experimental design method to correctly predict kinetics of cruciferin concentration and napin purity. The resulting model was validated with three different experiments and implemented in a multicriteria optimization tool to identify optimal conditions to achieve maximum napin purity at minimum enzymatic cost and production time. The results showed that the approach allowed optimization of the selective hydrolysis of cruciferins with the production of peptides with equivalent iron-chelating activity while meeting technical implementation constraints and reducing production costs. 

The peptides obtained in these optimized conditions should be further purified by a separation tool, such as Immobilized Metal Affinity Chromatography (IMAC), and could be identified by mass spectrometry. Further characterization in food models like oil-in-water emulsions could be of interest to evaluate their applicability in food safety. The bioavailability of chelated iron could also be studied to allow applications in human health as “micronutrient-rich foods” [45]. Finally, the antimicrobial properties of the purified napins produced under optimal conditions could also be studied on different microorganisms involved in food spoilage. This could help the sustainable valorization of rapeseed meal in food, cosmetic, or pharmaceutical industries.

## Figures and Tables

**Table 1:** Codification of operating condition parameters.

Level	T (°C)	E/S (g Enzyme/g Substrate)
−1	35	1/2000
0	45	1/285
1	55	1/154

**Table 2:** DoE modeling experiments, coded and real values.

Experience Name	T (°C)		E/S (g Enzyme/g Substrate)	
a	−0.75	37.5	−0.75	1/801
b	−0.75	37.5	0.75	1/174
c	0.75	52.5	−0.75	1/801

**Table 3:** Analysis of model results and regression coefficients of predicted model for kinetic parameter $k_{C}$ of cruciferin concentration.

	Variables	$\mathrm{Kinetic}\;\mathrm{Constant}\;k_{C}$
**Significant Coefficients**	Constant $\left(b_{0}\right)$	10.16
	$\mathrm{ES}\;\left(b_{1}\right)$	8.49
	T $\left(b_{2}\right)$	9.11
	ES× $T\;\left(b_{3}\right)$	9.76
	ES^2^ $\left(b_{4}\right)$	-
	T^2^ $\left(b_{5}\right)$	4.078
**Model analysis**	RSD	1.306
	R²	0.99
	Q²	0.96
	Model (*p*-value)	2.58 × 10^−6^

## Data Availability

Data is contained within the article.
